# Levels of stigma among community mental health staff in Guangzhou, China

**DOI:** 10.1186/s12888-014-0231-x

**Published:** 2014-08-13

**Authors:** Jie Li, Juan Li, Graham Thornicroft, Yuanguang Huang

**Affiliations:** Guangzhou Psychiatric Hospital, Guangzhou Medical University, 36# Mingxin Road, Liwan, Guangzhou, 510370 China; Health Service and Population Research Department, King’s College London, Institute of Psychiatry, De Crespigny, London, SE5 8AF UK

**Keywords:** Stigma, Mental health staff, Psychometric properties

## Abstract

**Background:**

Stigma and discrimination are widely experienced by people with mental illness, even in healthcare settings. The purposes of this study were to assess mental health stigma among community mental health staff in Guangzhou, China and in doing so also to assess the psychometric properties of the Reported and Intended Behaviour Scale (RIBS) - Chinese version.

**Methods:**

A cross-sectional survey was undertaken among 214 community mental health staff in Guangzhou from September to November, 2013. The Mental Health Knowledge Schedule (MAKS) and RIBS were administered together with the Mental Illness: Clinicians’ Attitudes Scale (MICA) to evaluate staff stigma from the perspective of knowledge, attitudes and behaviour.

**Results:**

The total scores of RIBS, MAKS and MICA were (11.97 ± 3.41), (16.80 ± 5.39) and (51.69 ± 6.94) respectively. Female staff members were more willing to contact people with mental illness than males (*t*(212) = −2.85,*P* = 0.005) and had more knowledge about mental illness (*t*(212) = −2.28,*P* = 0.024). The Chinese version of RIBS had good internal consistency (alpha = 0.82), test-retest reliability (*r* = 0.68,*P* < 0.001) and adequate convergent validity, as indicated by a significant negative correlation with the Chinese version of MICA(*r* = −0.43*, P* < 0.001).

**Conclusions:**

Our results show relatively high levels of stigma toward people with mental illness among community mental health staff in Guangzhou, China. There are slightly gender differences in discriminatory behaviours and stigma related knowledge of mental illness among community mental health staff, with female staff in general less stigmatising. Accordingly, anti-stigma programmes should be established among healthcare staff. In addition, the Chinese version of RIBS is a reliable, valid and acceptable measure which can be used to assess the willingness of participants to contact people with mental illness in future anti-stigma campaigns.

## Background

Increasing evidence suggests that the stigma associated with mental illness is a powerful global barrier to the provision of mental health care [1,2] and is widely experienced by people living with mental illness [3-7]. From a clinical perspective, stigma and the ensuing discriminatory behaviours, have a negative influence on the severity of symptoms [6,8-10], willingness to seek help [11-14], compliance with treatment [15], as well as clinical outcomes [6,9]. From a public health perceptive, stigma may have an adverse effect on the social function of people with mental illness [10,16-19], on employment [4,5], on risk of suicide [8,20], as well as constraining the development of mental health programs, with structural discrimination limiting service and research investment [13,21-25].

Based on these insights, the World Psychiatric Association (WPA) began an international programme “Open the Doors” to fight the stigma and discrimination because of schizophrenia. Since then, such local projects have been implemented in more than 20 countries, and involved about 200 different anti-stigma projects [26-29]. However, it is now clear that mental health service provider may themselves be in need of de-stigmatization. Many people with mental illness reported stigma experiences within the health care services perpetuated by health professionals [30-34]. Further there is some evidence to support the view that health care staff hold even more negative attitudes toward people with mental illness than the general population, for example in preferring to contact patients by phone than in person, because of perceptions of risk of violence [35].

Many countries are integrating mental health care into services for people with chronic conditions [21] in order to reduce the large treatment gap in mental health. China is going through a transition of emphasis from psychiatric hospitals to primary care settings so as to increase access to mental healthcare [36]. To better deliver mental health services, Guangzhou is exploring a new community service model from the perspective of public health rather than merely a clinical approach. It includes training staff, service provision to patients in the community, and assessment of training and service outcomes. We believe that reducing stigma will even further advance mental health services.

Thornicroft defined stigma as an overarching term that contains three elements: problems of knowledge (ignorance), problems of attitudes (prejudice), and problems of behaviour (discrimination) [37]. Most recent stigma intervention studies targeted at healthcare staff have focused upon staff attitudes [35,38]. Further, those studies which have investigated discriminatory behaviours have usually assessed participants in an imaginary context [37], for example using vignettes, which may be different from real life situations. In China, although there are some studies on stigma, most of these concentrate on patients and their relatives [39,40], and few focus on mental health staff, not to mention community staff, who undertake increasing treatment and management work and have significant influences on patients’ treatment adherence. In terms of measuring stigma related outcomes, the Reported and Intended Behaviour Scale (RIBS) was developed to assess reported (past) and intended (future) behavioural discrimination among respondents towards people with mental health problems [41].

### Aims

The aim of this study were to understand the full scope of stigma among community mental health staff in Guangzhou, China in terms of knowledge, attitude and behaviour for the first time, and in doing so to test the reliability, validity and acceptability of the Chinese version of the RIBS. We tested the primary hypothesize that people who hold more negative attitudes toward people with mental illness will have more discriminatory behaviours. In addition, we aimed to discover which demographic variables are associated with discrimination prior to designing an intervention study. According to previous research in other countries [42-44], we secondarily hypothesize that there are significant gender difference in stigma: female staff would be less discriminating toward people with mental illness than male.

## Methods

### Participants

All the community mental health staff in Guangzhou, China were invited to take part in the survey. Those who refused to join the study were excluded. A sub-sample of participants were also asked to be participants to establish the test-retest reliability of the RIBS. The survey was conducted in individual interviews from September, 2013 to November, 2013. The study protocol was approved by Research Ethics Committee of Guangzhou Psychiatric Hospital (Number 66, 2013) and written informed consent was obtained from each participant after the procedure had been fully explained.

### Instruments

A questionnaire was used to collect basic socio-demographic data for age, gender, level of education and marital status. The Reported and Intended Behaviour Scale (RIBS) is an 8-item scale used to assess mental health-related reported and intended behavioural discrimination among the general public. The original version of RIBS has been showed to be a psychometrically robust measure with strong internal consistency (α = 0.85) and test-retest reliability (*r* = 0.75) [41]. Items 1–4 only calculate the prevalence of behaviour. Items 5–8 were used for assessing the willingness to engage in the stated behaviour and rated on a 5-point scale anchored at 1 = totally disagree and 5 = totally agree. “Don’t know” was coded as neutral (i.e. 3). The total score was calculated by adding the response values for items 5–8 and can range from 4 to 20. A higher score indicates greater willingness to contact people with mental illness.

The stigma-related mental illness knowledge was assessed using Mental Health Knowledge Schedule (MAKS). Although MAKS was not developed to function as a scale, it was designed, to be used in conjunction with other attitude- and behaviour-related measures [45]. MAKS comprises 12 items. The first 6 items cover stigma-related mental health knowledge areas: help seeking, recognition, support, employment, treatment and recovery. For example, “Most people with mental health problems want to have paid employment”. Items 7–12 assess opinions about which condition are types of mental illness to help contextualize the responses to other items, for example, “Depression”. A 5-point Likert scale was used and response options range from 1 = totally disagree to 5 = totally agree. “Don’t know” was valued 3 for the purposes of determining a total score. The total score was calculated by adding the response values for only items 1–6 and can range from 6 to 30. A higher score indicates more knowledge.

The Mental Illness: Clinicians’ Attitudes Scale (MICA)-Chinese version was specially designed for assessing the level of stigmatizing attitudes to mental illness and psychiatric staff [46]. It contains 16 items. Each item is rated on a 6-point scale anchored at 1 = totally agree and 6 = totally disagree. Scores can range from 16 to 96 and a lower score indicates less stigma. The Chinese version of MICA has been reported to have strong internal consistency (α = 0.72) and test-retest reliability (*r* = 0.76) in a parallel study that we have conducted.

Participants were also asked to answer two additional items: “I would like to avoid contact people with mental illness” and “People with mental illness in a stable state could participate in social activities”. They were rated by 1 = totally agree to 6 = totally disagree. In addition, all participants were asked to comment on each scale about how far they understood the scale with a 6-point Likert scale, 1 = total incomprehension to 6 = total comprehension.

### Translation

Once permission was obtained from the original authors, RIBS and MAKS were translated into Chinese. Two professors of psychiatry, a doctoral candidate of anthropology and a postgraduate student of psychiatry contribute to the forward translation. And then, a senior psychiatrist who had worked for more than 10 years in the US completed the back-translation.

### Data analysis

Data were analyzed using SPSS v13. Statistical methods include a general description of quantitative data, *t*-testing for continuous variables, chi square test to compare categorical variables, and bivariate correlations between scores were assessed with Pearson correlation coefficient. An item analysis was conducted and Cronbach’s alpha was computed to determine the overall consistency of the scale. Item-total correlations were calculated along with Pearson correlation coefficient. Test-retest reliability of the scale was completed with a sub-sample of participants who consented to be contacted one week after they had completed the first survey. A Pearson correlation coefficient of the total scores before and after one week period was calculated to assess the test-retest reliability.

## Results

### Demographic characteristics

A response rate of 81.1% was achieved (214 of 264) and 79 participants consented to complete the scales one week later to establish the test-retest reliability. The participants’ socio-demographic characteristics are shown in Table 1. There was no significant difference between male and female participants in terms of age, education or marital status.

**Table 1:** **Demographic characteristics of the sample**

**Variable**		**N**	**%**
Age (n = 214)	Mean (S.D.) = 34.29 (8.56).	Range =22-60	
Gender	Male	113	52.8
	Female	101	47.2
Levels of education	Senior high school	17	7.9
	Undergraduate	189	88.3
	Postgraduate	8	3.7
Marital status	Single	67	31.3
	Married	147	68.7

### Stigma scores

The mean total score for the RIBS was 11.97 (SD = 3.41) and scores ranged from 4 to 20. Scores range from 8 to 29 for MAKS with a mean score of 16.80 (SD = 5.39). For MICA, scores ranged from 29 to 68 and the mean score was 51.69 (SD = 6.94).

### Responses frequencies for the additional items in the survey

For “I would like to avoid contact people with mental illness”, 34.3% (n = 214) replied as totally agree or agree, whereas, a total of 55.1% (n = 214) participants answered “totally agree or agree” when asked “People with mental illness in a stable state could participate social activities”. Tables 2 and 3 show responses to the last 4 RIBS and the first 6 MAKS items respectively.

**Table 2:** **Response frequencies for the first 4 items of RIBS (n,%)**

	**Yes**	**No**	**Don’t know**
1. Are you currently living with, or have you ever lived with, someone with a mental health problem?	34 (15.9)	174 (81.3)	6 (2.8)
2. Are you currently working with, or have you ever worked with someone with a mental health problem?	56 (26.2)	146 (68.2)	12 (5.6)
3. Do you currently have, or have you ever had, a neighbor with a mental health problem?	65 (30.4)	127 (59.3)	22 (10.3)
4. Do you currently have, or have you ever had, a close friend with a mental health problem?	31 (14.5)	169 (79.0)	14 (6.5)

**Table 3:** **Participants’ opinion on which condition is a type of mental illness for the MAKS (n,%)**

	**Totally agree**	**Slightly agree**	**Neither agree nor disagree**	**Slightly disagree**	**Disagree**	**Don’t know**
1. Depression	149 (69.6)	26 (12.1)	7 (3.3)	32 (15.0)	0	0
2. Stress	40 (18.7)	52 (24.3)	35 (16.4)	42 (19.6)	35 (16.4)	10 (4.7)
3. Schizophrenia	203 (94.8)	3 (1.4)	1 (0.5)	7 (3.3)	0	0
4. Bipolar disorder (manic depression)	200 (93.4)	3 (1.4)	1 (0.5)	9 (4.2)	0	1 (0.5)
5. Drug addiction	58 (27.1)	35 (16.4)	17 (7.9)	41 (19.2)	56 (26.2)	7 (3.3)
6. Grief	66 (30.8)	39 (18.2)	29 (13.6)	33 (15.4)	39 (18.2)	8 (3.7)

### RIBS total score and socio-demographic variables

#### 1) Comparison by gender

The mean score was 11.35 (SD = 3.36)for males and 12.66 (SD = 3.35)for females and the difference was statistically significant (*t*(212) = −2.85, *P* = 0.005). In addition, the difference also remained when comparing the scores of MAKS between male and female participants (16.02 vs. 17.68, *t*(212) = −2.28,*P* = 0.024).

#### 2) Comparison by age

There was no difference between the 22–35 years old and 36–60 years old groups in terms of RIBS scores (*t*(212) = −0.34,*P* = 0.734). The mean scores were 11.91 (SD = 3.48) and 12.08 (SD = 3.30)respectively.

#### 3) Correlations among total scores of RIBS, MAKS and MICA

The MICA scores had negative correlations with RIBS (*r* = −0.43,*P* < 0.001) and MAKS (*r* = −0.17, *P* = 0.011). There was a positive correlation between RIBS and MAKS (*r* = 0.30, *P* < 0.001).

#### 4) Correlations between reported and intended behaviours in terms of RIBS

According to the responses (Yes or No) to the first 4 items in RIBS, the participants were divided into different groups. We examined the difference between each group in terms of the second set of 4 items in RIBS. Participants who “currently living with, or have ever lived with, someone with a mental health problem” scored higher on the item “In the future, I would be willing to continue a relationship with a friend who developed a mental health problem” (3.32 VS. 2.80, *t*(206) = 2.29, *P* = 0.023).

### Psychometric evaluation of the RIBS

#### 1) Reliability

Cronbach’s alpha among items 5–8 of the RIBS was 0.82 and removal of any item did not increase the α value to greater than 0.85, indicating good internal consistency. The item-total correlations were ≥0.74 for all the items. Test-retest reliability separated by one week was 0.68, *P* < 0.001, n = 79). This indicates that the scale is stable over this time period.

#### 2) Validity

Consistent with our main hypothesis, more negative attitudes to people with mental illness were associated with greater social distance. We found that there was a significant negative correlation between the total score of RIBS and that of the MICA (*r* = −0.43*, P* < 0.001). There was also a significant positive correlation between the total score of RIBS and that of the MAKS(*r* = 0.30*, P* < 0.001).

#### 3) Feasibility and acceptability

There are only 8 items of RIBS, which is very feasible for most conceivable uses of the scale. The RIBS scale is of good acceptability since there were no missing data. Above all, there was no ceiling effect or floor effect since participants tended to use the full range of the response options. Overall, a total of 87.8% of participants indicated that they completely understood or understood the RIBS scale.

## Discussion

To our knowledge, this is the first study to investigate the levels of stigma among community mental health staff in China. Our results showed relatively high levels of stigma toward people with mental illness among community mental health staff in Guangzhou. The mean score were 11.97, 16.8 and 51.69 for RIBS,MAKS and MICA respectively. To put this in context, Henderson *et al.* use RIBS and MAKS to evaluate the impact on the general population of England’s Time to Change program, and their results showed that the mean scores were 14.5 for RIBS and 21.2 for MAKS [47]. Moreover, Ye Rong *et al.* researched attitudes toward depression among medical students and their results showed that the mean score was 43.51 for MICA [38]. We can conclude that the community mental health staff in Guangzhou not only have low levels of stigma related knowledge of mental illness but also less likely to contact people with mental illness and held a relatively negative attitude toward them.

Although these differences above could be partly due to the low proportion of female participants, which was in line with our finding that females are more willing to contact people with mental illness. However, the responses to the additional items strongly support the need for anti-stigma interventions among mental health staff in Guangzhou. For the response to the additional item “I would like to avoid contact with patients with mental illness”, more than one-third of the participants reported totally agree or agree, showing us a rather pessimistic picture.

In support of our hypothesis, there are clear gender differences in terms of behavioural discrimination and knowledge related to mental illness, which are also in line with previous studies [38,48,49], which have investigated, for example, teenagers or medical students. But the mean score of RIBS were 11.35 and 12.66 for males and females respectively. For the MAKS, the gender difference was even smaller. The slight difference make it difficult to draw a conclusion. In addition, evidence from other study also provided rather mixed results [50].Wang *et al.* indicated that women had significantly more negative overall implicit attitudes, especially negative cognition and beliefs, toward mental illness than men [51], and we propose that future study may well explore levels of stigma measured using implicit measures.

Social contact can be an effective intervention for reducing social exclusion in anti-sigma campaigns [27,29]. Consistent with this, we have found that participants who reported “Yes” to item “Do you currently live with, or have ever lived with, someone with a mental health problem?” showed more willingness to continue a relationship with a friend who developed a mental health problem than those reported “No”. A recent meta-analysis [52] showed that contact-based strategies led to stronger outcomes than educational methods. And this is why many anti-stigma campaigns use a method of social contact with persons with mental illness among targeted groups to improve attitudes toward people with mental illness [26,27,29].

In this study, participants put schizophrenia, bipolar disorder and depression in the first three positions which they preferred to perceive as mental illness based on their stereotypes. As previous studies have also found, people with schizophrenia are more likely to be perceived as violent and unpredictable than people with other mental health problems [53]. This can lead to a high level of discrimination against people with schizophrenia in healthcare settings. On the other hand, we found that the proportion of negative attitudes toward people with depression was lower than for schizophrenia. In recent years, some notable persons’ experience of depression has improved the perception of the illness in public and the tolerance toward depression in China. In addition people are more inclined to attribute depression to stress, than to associate stress with schizophrenia.

There are considerable cultural variations in how stigma is manifested [54,55]. Members of Asian cultures may express greater stigma compared to Western cultural counterparts [55]. In China, which is a society/community-orientated country and takes “face” (mianzi) as central to social identity, having anything to do with mental illness means being faceless or shamed [51,56]. Therefore, it makes great significance to have a clear picture of levels of stigma among mental health staff, to assess in the context of quality of care. For the purpose, assessment instruments with strong psychometric properties are essential.

Our study found that RIBS was a brief and feasible instrument that can be used to assess the mental health staff’s willingness to contact people with mental illness. It also revealed that the Chinese version of the scale was psychometrically robust, fully meeting reliability, validity, feasibility and acceptability criteria. The assessment of reported and intended behaviour together is significant as it allows the observation of both types of information and their relationship over time in the study population. In brief, the RIBS scale can be applied to measure healthcare professionals’ and the general population’s behavioural discrimination toward people with mental illness before and after anti-stigma interventions in China.

There are some limitations to the study that should be noted. Since the study was a cross-sectional design, it is cannot be used to make inferences about causality. Also we only investigated community mental health staff, so no comparison is possible with, for example, with hospital staff.

## Conclusions

Our results showed relatively high levels of stigma toward people with mental illness among community mental health staff in Guangzhou, China. The three elements of stigma are all present among community mental health staff in Guangzhou, problems of knowledge (ignorance), problems of attitudes (prejudice) and problems of behaviour (discrimination). Considering that more and more people with mental illness will receive treatment in the community in China, it is important to carry out anti-stigma programs among community mental health staff, to improve the quality of care provided. In addition, the Chinese version of RIBS could be used as an effective instrument to evaluate the impact on such campaigns.
